# A midline abdominal incision: can it be that dangerous? A huge umbilical vein in a cirrhotic patient

**DOI:** 10.11604/pamj.2022.43.178.38187

**Published:** 2022-12-06

**Authors:** Mohamed Al Amine El Mouden, Anas Ahllat

**Affiliations:** 1 Department of General Surgery, University Hospital Center, Tangier, Morocco

**Keywords:** Umbilical vein, portal hypertension, midline abdominal incision

## Image in medicine

The umbilical vein transport oxygenated blood from the placenta to the fetus. It travels through the umbilical cord, which disappears at birth. It divides in two, one part going to the liver, the other one flowing into the inferior vena cava through the ductus venosus. During labor, uterine contractions cause an increase in fetal arterial pressure, which results in a decrease in the flow of para umbilical veins. A decrease in intrahepatic blood flow in the case of liver cirrhosis increases resistance in the portal vein, resulting in portal hypertension. In order to combat portal hypertension, portosystemic shunts develop in four directions: superior (left gastric vein), inferior (via hemorrhoidal veins), posterior (left renal vein), and anterior with repermeabilization of the umbilical vein and abdominal wall veins giving Cruveilhier-Baumgarten syndrome. We report the case of a 41-year-old patient followed for a hepatic cirrhosis without obvious etiology classified as child A with portal hypertension responsible for an important splenomegaly and a hypersplenism associated with a repermeabilization of the umbilical vein, operated in our general surgery department at the University Hospital of Tangier, for a non-metastatic neuroendocrine carcinoma of the gastric antrum having benefited from an antrectomy with lymph node dissection. It should be noted that the incision was in the midline above the umbilicus strict and very careful because of the risk of damaging the umbilical vein which was of large caliber and can cause a cataclysmic and uncontrollable hemorrhage. We present the impressive image of the umbilical vein in this patient.

**Figure 1:**
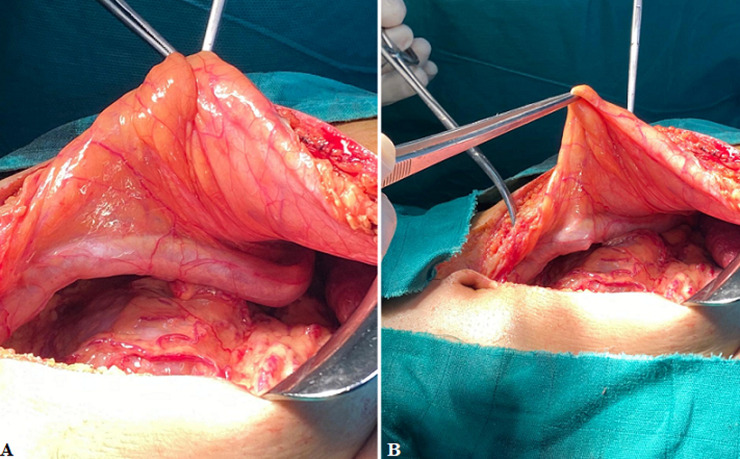
A) midline abdominal incision with visualization of a huge umbilical vein; B) midline abdominal incision respecting the umbilicus

